# Mutant MAPT Induces rDNA Transcriptional Hyperactivation and Nucleolar Stress in Cellular Models

**DOI:** 10.21203/rs.3.rs-7713987/v1

**Published:** 2025-10-05

**Authors:** Zaid Muhammad, Yan Gu, Suleiman H. Kwairanga, Laura J. Bailey, Amna Khan, Mohammad Nasser, Dana Aljarrah, Charles Arber, Selina Wray, Louise C. Serpell, Celeste M. Karch, Mahmoud B. Maina

**Affiliations:** Sussex Neuroscience, School of Life Sciences, University of Sussex, Brighton, UK; Biomedical Science Research and Training Centre, Yobe State University, Damaturu, Nigeria; Biomedical Science Research and Training Centre, Yobe State University, Damaturu, Nigeria; Sussex Neuroscience, School of Life Sciences, University of Sussex, Brighton, UK; Biomedical Science Research and Training Centre, Yobe State University, Damaturu, Nigeria; Faculty of Medical and Health Science, Newgate University Minna, Nigeria; Biomedical Science Research and Training Centre, Yobe State University, Damaturu, Nigeria; Biomedical Science Research and Training Centre, Yobe State University, Damaturu, Nigeria; Biomedical Science Research and Training Centre, Yobe State University, Damaturu, Nigeria; Department of Neurodegenerative Disease, UCL Queen Square Institute of Neurology, London, UK; Department of Neurodegenerative Disease, UCL Queen Square Institute of Neurology, London, UK; Biomedical Science Research and Training Centre, Yobe State University, Damaturu, Nigeria; Department of Psychiatry, Washington University in St. Louis, St. Louis, Missouri, USA; Sussex Neuroscience, School of Life Sciences, University of Sussex, Brighton, UK; Biomedical Science Research and Training Centre, Yobe State University, Damaturu, Nigeria; Faculty of Medical and Health Science, Newgate University Minna, Nigeria

## Abstract

Tau is traditionally known for its role in microtubule stabilization, with its pathological aggregation central to tauopathies such as Alzheimer’s disease (AD) and frontotemporal dementia (FTD). Recent evidence suggests that tau also plays important nuclear and nucleolar roles, yet the implications of tau pathology on nucleolar function remain poorly understood. Here, we show that tau localises to the nucleolus in both differentiated SH-SY5Y cells and iPSC-derived neurons, and accumulates upon expression of disease-associated MAPT mutations (P301S, S305N, and IVS 10 + 16). Using high-content imaging, we demonstrate that mutant tau expression leads to structural expansion of the nucleus and nucleolus, with upregulation of key markers from all three nucleolar sub-compartments, indicating increased in nucleolar activity. qPCR and nucleolar RNA-selective dye staining confirmed increased rDNA transcription and rRNA processing, suggesting that mutant tau drives elevated nucleolar biosynthetic output. This hyperactivation is accompanied by hallmarks of nucleolar stress and apoptosis, including p53 stabilisation, caspase 3/7 activation, and TUNEL positivity. These findings identify nucleolar dysfunction as a downstream consequence of mutant tau expression and highlight disruption of nucleolar homeostasis as a potential contributor to tau-mediated neurotoxicity in MAPT-linked FTD.

## Introduction

Since its discovery in 1975 as a microtubule-stabilising protein ^1^, the microtubule-associated protein tau (MAPT) has become one of the most studied proteins in neurodegeneration. Tau is a major component of neurofibrillary tangles, one of the defining hallmarks of Alzheimer’s disease (AD), alongside amyloid-β plaques ^2–4^. Beyond AD, tau is implicated in a broader group of neurodegenerative diseases known as tauopathies, which include corticobasal degeneration, frontotemporal lobar degeneration, pick’s disease, and progressive supranuclear palsy ^5^. Additionally, mutations in the *MAPT* gene can directly cause neurodegeneration, as observed in familial forms of frontotemporal lobar degeneration ^6,7^.

While historically considered a cytoplasmic protein, tau has been found in multiple subcellular compartments including the nucleus where it plays regulatory functions on chromatin compaction ^8–12^, DNA integrity ^13,14^ and RNA metabolism ^14–16^. These functions are particularly relevant in instances of cellular stress and disease progression ^17,18^. Tau has also been found in the nucleolus ^19–22^, a membrane-less subnuclear organelle responsible for ribosomal RNA (rRNA) transcription and processing, and the assembly of ribosomal subunits. Indeed, we have previously described the localization of tau within the nucleolus in both cell lines and human brain tissue, where it is associated with the nucleolar-remodelling complex, a previously unreported association ^16^.

The primary role of the nucleolus is to transcribe ribosomal DNA (rDNA) into a 47S precursor, which is processed into 45S pre-rRNA and subsequently cleaved to produce the mature 18S, 5.8S, and 28S ribosomal RNAs (rRNAs). These rRNAs then assemble with ribosomal proteins to form functional ribosomes ^23^. Given its pivotal role in cellular protein synthesis, this process is energetically demanding and tightly regulated, positioning the nucleolus as a key sensor of cellular stress ^23^. Under conditions such as transcriptional dysregulation, DNA damage, oxidative stress, or proteotoxic insults, a phenomenon known as nucleolar stress is triggered – a concept first described in the 1980s ^24^. This response is typically marked by alterations in nucleolar size, number, and morphology, rDNA transcription dysregulation, and accumulation of free ribosomal proteins (r-proteins), such as RPL11, in the nucleoplasm. It is also accompanied by redistribution or degradation of key nucleolar factors including Upstream Binding Factor (UBF), Fibrillarin (FBL), and Nucleophosmin (NPM) ^25–27^. As a result, some of these proteins e.g. NPM and RPL11, bind and inhibit the ubiquitin ligase MDM2, which normally ubiquitinates p53 and targets it to the proteasome under unstressed conditions. Consequently, p53 becomes liberated, resulting in the activation of the apoptotic pathway ^28,29^.

Recent studies suggest that nucleolar dysfunction contributes to neurodegenerative disease by activating nucleolar stress response which could impair ribosome production, induce proteostasis imbalance and activate apoptotic pathways ^27,30–38^. Given the localisation of tau in the nucleolus and role of tau pathology in neurodegenerative diseases, this has led to a growing interest about the interaction between nuclear tau and nucleolar function ^16,39,40^.

Several MAPT mutations, such as P301S and S305N, as well as splicing variants like IVS 10 + 16, result in frontotemporal dementia (FTD) ^6,41–44^. Many of these mutations alter tau isoform balance by enhancing 4R-tau isoform expression and thus triggering a neurotoxic cascade ^45,46^. While much is known about the effects of MAPT mutations on tau aggregation and microtubule dynamics, their influence on nucleolar function remains unknown despite tau’s emerging role in the nucleolus. We have previously established that nucleolar non-phosphorylated tau (nP-Tau) becomes redistributed to the nucleoplasm, similar to canonical nucleolar proteins like UBF, FBL and NPM, under nucleolar stress conditions ^40^. We further showed that under normal conditions, tau regulates rDNA transcription ^16^. These observations raise critical questions: do MAPT mutations alter nucleolar function and the ribosomal synthesis pathways?

To address this, we used SH-SY5Y cells with tau overexpression under the control of a tetracycline-inducible promoter, alongside iPSC-derived neurons carrying different disease-relevant MAPT mutations, to examine the impact of tau dysfunction on nucleolar function. Using high-content imaging and transcriptomic analysis, we show that MAPT mutations enhance nucleolar volume and rRNA synthesis, alongside nucleolar stress-induced p53 signalling, and apoptosis. Our findings support a model in which mutant tau drives rDNA hyperactivation, contributing to nucleolar stress and downstream neurotoxicity. This highlights the nucleolus as a previously underappreciated site of vulnerability in tauopathies and a potential contributor to disease pathogenesis.

## Methods

### Cell Culture

SH-SY5Y neuroblastoma cell lines obtained from Prof. Luc Buée (Université de Lille, France) expressing tetracycline-inducible human 4-repeat (4R) tau with P301S or S305N mutations or empty vector (EV) were used in the experiments ^47^. Cells were cultured in Advanced DMEM (1X) supplemented with 10% tetracycline-free fetal calf serum (tet-free FCS), 1% non-essential amino acids, 1% L-glutamine, and 0.5% penicillin-streptomycin. The cultures were maintained at 37°C in a humidified incubator with 5% CO_2_.

### Retinoic Acid Differentiation and Tetracycline Induction

Cells were partially differentiated using 10 μM retinoic acid (RA) (Sigma-Aldrich, Cat# R2625) in Advanced DMEM (1X) supplemented with 1% tet-free FCS, 1% L-glutamine, 1% non-essential amino acids, and 0.5% penicillin-streptomycin. Differentiation was performed over three days under standard culture conditions (37°C, 5% CO_2_). To induce transgene expression, both mutant (P301S and S305N) and EV lines were treated with 1 μg/mL tetracycline in differentiation medium containing 10 μM RA after the initial three days of RA differentiation. Tetracycline induction was carried out for timepoints of 1 hour and 48 hours in the absence of light to prevent RA degradation.

### Induced Pluripotent Stem Cell (iPSC) Culture and Differentiation

Human induced pluripotent stem cell (iPSC) lines carrying disease-associated *MAPT* mutations were used to model tauopathy-related cellular phenotypes. A CRISPR-edited iPSC line harboring a heterozygous *MAPT* P301S mutation (F0510.2d3B07) and its isogenic control (F0510.2d2E07) (both derived from a donor with a *MAPT* P301L/WT background) were generously provided by Professor Celeste Karch (Washington University in St. Louis, USA). A second iPSC line carrying the IVS 10 + 16 splice-site mutation in *MAPT* and a donor-matched, non-isogenic control line were kindly provided by Professor Selina Wray (University College London, UK) ^48^. All the iPSC lines were maintained in mTeSR^™^ Plus medium (STEMCELL TECHNOLOGIES, Cat# 100-0276) under standard conditions: 37°C, 5% CO_2_, and > 90% relative humidity. Cells were cultured on matrigel-coated plates and monitored daily for morphology. Colonies displaying smooth edges and compact morphology were expanded for a few days before being used for neural progenitor cell (NPC) induction. To induce iPSCs to neuronal fate, they were passaged using ReLeSR^™^ (STEMCELL TECHNOLOGIES, Cat# 100-0484) and dissociated into a single-cell suspension in Neural Induction Medium supplemented with SMADi (STEMCELL TECHNOLOGIES, Cat# 08581). Cells were plated at a density of 1.2 × 10^6^ cells/mL into a freshly prepared matrigel-coated 6-well plate. The induction medium was supplemented with 10 μM ROCK inhibitor (Y-27632; STEMCELL TECHNOLOGIES, Cat# 72302) during initial plating to enhance cell survival and attachment. Media was refreshed daily, and cells were passage between days 6 and 9, depending on confluency according to the manufacturer’s protocol. Following three passages, the resulting cultures displayed rosette-like morphology and were positive for NPC markers Nestin and Pax6, as confirmed by immunostaining. These cells were then maintained and expanded in Neural Progenitor Basal Medium (STEMCELL TECHNOLOGIES, Catalog # 05833) and used for cryopreservation and subsequent differentiation steps. For neuronal differentiation, 6-well plates were pre-coated with matrigel. NPCs were passaged using Accutase (STEMCELL TECHNOLOGIES, Cat# 07922) and dissociated into single cells. Cells were then resuspended in Forebrain Neuronal Differentiation Medium (STEMCELL TECHNOLOGIES, Cat# 08600) supplemented with CultureOne supplement (ThermoFisher, Cat# A3320201). Cells were seeded at a density of 1 × 10^6^ cells/mL into the prepared wells and medium replaced daily to maintain optimal differentiation conditions. At day seven, cells were passaged and seeded for forebrain neuronal maturation in a 6-well (for RNA extraction) and 96-well plates (for imaging) pre-coated with poly-L-ornithine (Sigma-Aldrich, Cat# P4957) and laminin (StemCell Technologies, Cat# 200 – 0117). Differentiated NPCs were passaged using Accutase and dissociated into single cells. IVS 10 + 16 and donor control iPSCs were differentiated for 50 days, while the P301S and isogenic control iPSCs were differentiated for 75 days, measured from the onset of NPC induction. These time points were selected to align with the earliest window of robust MAPT expression in each model, as IVS 10 + 16 neurons begin expressing tau earlier than P301S lines.

### Immunofluorescence (IF) Labelling

On the final day of culture, cells were fixed and stained for immunofluorescence analysis. Culture medium was aspirated, and cells were washed briefly with phosphate-buffered saline (PBS) before being fixed with 4% paraformaldehyde (PFA) for 15 minutes at room temperature. Following fixation, cells were washed three times with PBS and permeabilized using 0.5% Triton X-100 for 15 minutes. After another three PBS washes, cells were blocked in 4% bovine serum albumin (BSA) in PBS with 0.2% Tween-20 (PBST) for 45 minutes at room temperature. Cells were then incubated with primary antibodies (listed in Table 1) diluted in blocking solution for 45 minutes. After three PBST washes, cells were incubated with fluorescently conjugated secondary antibodies for an additional 45 minutes. Following another three washes, cells were stained with DAPI for 30 minutes to label nuclei. Finally, cells were washed three times and stored at 4°C in PBS until imaging.

### Functional Dye-Based Assays for Apoptosis, RNA, and Protein Synthesis

In addition to immunofluorescence antibody labelling, a range of functional assays were employed to assess nucleolar RNA abundance, protein synthesis, DNA fragmentation, and caspase activation, using dye-based detection kits. These were performed according to manufacturer protocols and integrated into the high-content imaging workflow. Apoptotic signaling was assessed using the CellEvent^™^ Caspase-3/7 Green ReadyProbes^™^ Reagent (ThermoFisher, Cat# R37111) for detection of activated caspase-3/7. A no-wash protocol was used to preserve fragile apoptotic cells, and staining was performed on live cells and used for high-content imaging. DNA fragmentation was visualized using the Click-iT^™^ Plus TUNEL Assay Kit (ThermoFisher, Cat# C10617), which incorporates modified dUTP into 3’-OH DNA ends during strand breaks, followed by a copper-catalyzed azide–alkyne click reaction for Alexa Fluor^™^ 488 fluorescent detection. Assays were conducted on fixed samples following the manufacturer’s protocol. Nucleolar RNA abundance was measured using the NUCLEOLAR-ID^®^ Green Detection Kit (Enzo Life Sciences, Cat# ENZ-51009), which contains a selective dye for nucleolar RNA structures. Cells were incubated with the staining reagent according to the manufacturer’s instructions prior to imaging. Global nascent protein synthesis was analyzed using the Click-iT^™^ Plus OPP Alexa Fluor^™^ 488 Protein Synthesis Assay Kit (ThermoFisher, Cat# C10428). This method is based on incorporation of O-propargyl-puromycin (OPP) into newly synthesized proteins, followed by fluorescent detection via a copper-catalyzed click chemistry reaction with Alexa Fluor azide. Labeling was performed on fixed cells.

### Image Acquisition and quantitative Analysis

Imaging was performed using the PerkinElmer Operetta CLS High-Content Scanning System (Model HH1600) in confocal mode using 40x water objective. Image acquisition and preliminary analysis were conducted using Harmony^®^ software version 4.9. Fluorescence intensities and morphological parameters were initially quantified using custom Harmony analysis protocols, and the data were exported as spreadsheets for further processing. Quantified data were then integrated in three dimensions using in-house scripts developed in Microsoft Excel with Visual Basic for Applications (VBA). Two separate analytical pipelines were applied: cyto-nuclear analysis and foci analysis.

### Cyto-Nuclear Analysis

DAPI-stained nuclei were segmented on each axial (z-stack) section based on size and fluorescence intensity. These nuclear masks were used to extract mean fluorescence intensities from the DAPI, Alexa Fluor 488, and Alexa Fluor 568 channels. The nuclear mask was also applied to define cytoplasmic boundaries, allowing quantification of Alexa Fluor 488 and 568 signals in the cytoplasmic compartment. 3D reconstruction was performed using a nearest-neighbour algorithm across axial sections, allowing volumetric integration of signal intensities. The resulting nuclear fluorescence volumes were averaged across all segmented cells within each well.

### Foci Analysis

Nucleolar signals in experiments labelled for nucleolar markers e.g. UBF, FBL and NPM, were quantified using foci quantification. This was initiated from the same nuclear segmentation used in cyto-nuclear analysis. Nuclear masks, along with predefined parameters for foci size and brightness, were applied to detect fluorescent foci in all axial sections. Detected foci were exported into Excel and integrated in 3D using the nearest-neighbour algorithm. Each focus was registered to its host nucleus, and total foci counts were averaged on a per-well basis. Fluorescence intensities from both foci and nuclei were also combined across all imaging fields and z-sections for each well.

### RNA Extraction, cDNA Synthesis, and qPCR

At the end of the culture period, cells were collected and pelleted by centrifugation. Total RNA was extracted using the Monarch Total RNA Miniprep Kit (New England Biolabs), following the manufacturer’s protocol. RNA concentration and purity were determined using a NanoDrop 2000 spectrophotometer (Thermo Fisher Scientific). Complementary DNA (cDNA) was synthesized from total RNA using the SuperScript^™^ VILO^™^ cDNA Synthesis Kit (Thermo Fisher Scientific), according to the manufacturer’s instructions. The thermal cycling conditions for cDNA synthesis were as follows: 25°C for 10 minutes, 42°C for 60 minutes, and 85°C for 5 minutes. Gene expression was measured by quantitative PCR (qPCR) using TaqMan^™^ Fast Advanced Master Mix (Applied Biosystems) and gene-specific TaqMan^™^ probes. Reactions were run on a Roche LightCycler^®^ 480 II system. The qPCR cycling protocol consisted of 50°C for 2 minutes (UNG incubation). 95°C for 20 seconds (enzyme activation), followed by 45 cycles of 95°C for 15 seconds (denaturation) and 60°C for 30 seconds (annealing/extension). Probes used for target gene amplification are listed in table 2:

### Statistical Analysis and Visualization

All statistical analyses and data visualizations were performed using Python (v3.11) within the Google Colab environment, leveraging libraries including scipy, statsmodels, seaborn, and matplotlib. Prior to conducting hypothesis testing, the normality of each dataset was assessed using the Shapiro–Wilk test. For non-normally distributed data, as indicated by Shapiro–Wilk p-values (e.g., p < 0.05), Mann–Whitney U test was used. In cases where data were normally distributed and met assumptions of homogeneity of variance (verified using Levene’s test), unpaired two-tailed t-tests were employed. A significance threshold of *p* < 0.05 was considered statistically significant. Significance annotations were reported using the following convention: ns: p > 0.05; *: p ≤ 0.05; **: p ≤ 0.01; ***: p ≤ 0.001; ****: p ≤ 0.0001. For qPCR analysis, relative gene expression was calculated using the ΔΔCt (delta delta Ct) method, with β-actin (*ACTB*) used as the primary reference housekeeping gene. Expression normalization was further validated using an independent housekeeping gene (RPS27) to ensure consistency. All qPCR data are reported as fold change relative to control conditions, and statistical comparisons were performed on the ΔCt values. High-content imaging datasets, which often included thousands of cellular measurements per condition (e.g., > 5000 nuclei in some experiments), were analyzed at the population level with appropriate per-cell statistical aggregation. All graphical summaries were visualized as box plots to highlight data dispersion, medians, and outliers.

## Results

### Mutant Tau Induces Structural Expansion of both Nuclear and Nucleolar Compartments in Differentiated SH-SY5Y Cells

**Figure 1:**
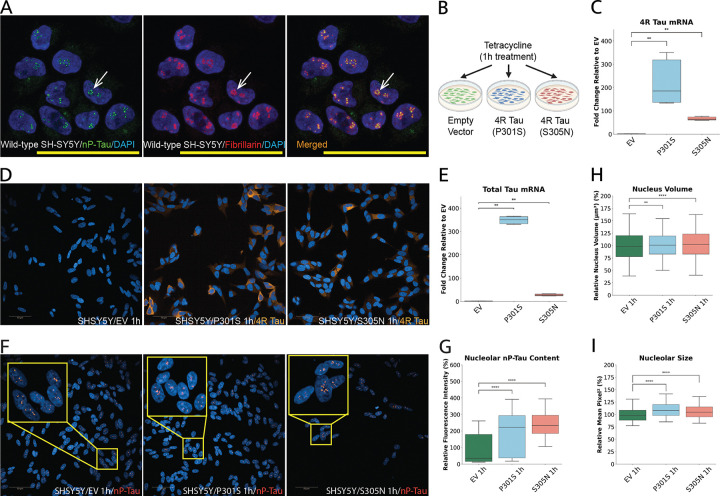
Mutant MAPT Expression Increases Nuclear and Nucleolar Volumes in SH-SY5Y Cells. (A) Immunofluorescence images showing colocalisation of non-phosphorylated tau (nP-Tau, green) with the nucleolar marker fibrillarin (FBL, red) in wild-type SH-SY5Y cells treated with retinoic acid (RA). DAPI (blue) marks nuclei. Arrows highlight nucleolar localisation of nP-Tau. (B) Schematic overview of the experimental set-up using a tetracycline-inducible system to express 4-repeat (4R) tau isoforms (P301S or S305N) or empty vector (EV) in RA-treated SH-SY5Y cells, following 1-hour tetracycline induction. (C) qPCR analysis confirming induced expression of MAPT 4R transcripts in P301S and S305N cells compared to EV, normalised to β-actin and RSP27, and expressed as fold change relative to EV. (D) Representative fluorescence images confirming robust 4R tau protein expression in mutant cells using a 4R isoform-specific antibody. (E) Total MAPT transcript levels (all isoforms) were also significantly increased in mutant cells, aligning with the 4R-specific findings. (F) Representative images and insets showing nucleolar localization of nP-Tau in EV, S305N, and P301S cells. (G) Quantification reveals significantly increased nucleolar nP-Tau content in mutant lines. High-content morphometric analyses show significantly increased nuclear volumes (H) and nucleolar sizes (I) in P301S and S305N cells relative to EV. Data represent mean ± SEM from ≥3 independent experiments, with ≥2,000 cells analyzed per condition and per experiment in high-content imaging experiments. Statistical comparisons were performed using two-sided Mann–Whitney U test with Bonferroni correction, following Shapiro–Wilk normality testing. *P < 0.05, **P < 0.01, ***P < 0.001, ***P < 0.0001. Scale bars: 10 μm (A); 50 μm (D, F).

There is a growing interest in the non-canonical roles of tau beyond its traditional function of binding to microtubules ^39,49^. We previously reported that tau localizes to the nucleolus in both differentiated and undifferentiated SH-SY5Y cells, as well as in human brain neurons, where it is associated with the nucleolar remodelling complex ^16^. Here, we began by confirming the colocalization of non-phosphorylated tau (nP-Tau) with the nucleolar marker FBL in the nucleolus of wild-type SH-SY5Y cells that were differentiated for three days via retinoic acid (RA) treatment (Fig. 1A). This short-term RA treatment results in a population of cells with arrested cell cycle and exhibiting neuronal characteristics ^50^.

To investigate whether FTD-linked *MAPT* mutations affect the nucleolar functions of tau, we used a tetracycline-inducible SH-SY5Y cell model developed by the Buée lab that expresses 4-repeat (4R) tau harbouring the P301S or S305N mutations, or an empty vector (EV) control ^47^ (Fig. 1B). In this system, the addition of tetracycline in the culture media results in the activation of the tau transgene, leading to robust induction of 4R tau expression detectable within 1 hour, as confirmed by qPCR (Fig. 1C). Thus, all subsequent experiments were conducted on RA-treated SH-SY5Y cells following 1-hour tetracycline induction unless otherwise noted. Importantly, the robust 4R tau expression in these mutant lines makes the use of a 4R isoform-specific probe a reliable measure of transgene expression, as confirmed by elevated 4R tau protein in both P301S and S305N lines compared to EV controls using high-content imaging (Fig. 1D). To complement this, we also performed qPCR using a total *MAPT* probe (detecting all isoforms) and found that both mutant lines exhibited increased overall tau expression, consistent with selective induction of 4R tau (Fig. 1E). Notably, the P301S-expressing cells consistently showed higher levels of both 4R tau and total *MAPT* transcripts than S305N, potentially reflecting differences in transgene integration or expression efficiency.

In previous studies, we demonstrated that nP-Tau behaves like typical nucleolar proteins. it becomes redistributed to the nucleoplasm upon induction of the nucleolar stress response and influences nucleolar function when knocked down ^16,40^. Consequently, we investigated the distribution of nP-Tau after expressing the P301S and S305N mutations (Fig. 1F). High-content imaging analysis revealed a significant accumulation of nP-Tau in the nucleolus of the P301S and S305N lines compared to the EV (Fig. 1G). Specifically, we observed an approximate 100% increase in nucleolar mean intensity in P301S cells and a ~ 140% increase in S305N cells, corresponding to ~ 2-fold and ~ 2.4-fold elevations, respectively. A similar increase in nP-Tau was observed in cells under sustained mutant tau expression for 48 hours (Supp. Figure 1A, B). These results suggest that mutant tau alters nP-Tau distribution, leading to its enhanced accumulation within the nucleolus.

The images of cells from these experiments consistently showed an increase in nuclear size, as indicated by DAPI staining. Thus, we utilized the DAPI-based nuclear segmentation to quantify the nuclear volume of the cells, revealing that the P301S and S305N lines exhibited a steady but moderate increase in mean nuclear volume of approximately 2.5% and 2.7%, respectively, compared to the control (EV) (Fig. 1H). After 48 hours of continuous mutant tau expression, this increase in nuclear volume became substantially more pronounced, with P301S and S305N cells showing a ~ 15% and ~ 13% increase, respectively, relative to EV (Supp. Figure 1C). Considering tau’s role in the nucleolus ^16,40^ and the observed accumulation of nP-Tau, we assessed nucleolar size using fibrillarin-based segmentation to investigate whether the MAPT mutations affect nucleolar structure. This analysis indicated that mutant tau expression significantly increases nucleolar size, with about a 10% increase in the P301S line and a 6% increase in the S305N line (Fig. 1I). At 48 hours post-induction, the nucleolar size exhibited an even more pronounced expansion, with P301S and S305N cells showing an ~ 12% and ~ 16% increase, respectively, compared to EV controls, suggesting that sustained *MAPT* mutation expression progressively enhances nucleolar remodelling (Supp. Figure 1D).

Overall, these findings demonstrate that short-term induction of disease-associated MAPT variants in differentiated SH-SY5Y cells results in pronounced nucleolar accumulation of nP-Tau, alongside structural expansion of both the nucleus and nucleolus. This structural remodelling emerges as an early phenotype that becomes more pronounced with sustained mutant tau expression. While the effects of the P301S and S305N mutations differed modestly in magnitude, both exhibited consistent effects across all structural parameters, indicating that mutant tau, irrespective of mutation type, exerts a shared upstream influence on nucleolar architecture and function.

### Mutant Tau Drives rDNA Transcriptional Hyperactivation in Differentiated SH-SY5Y Cells

**Figure 2:**
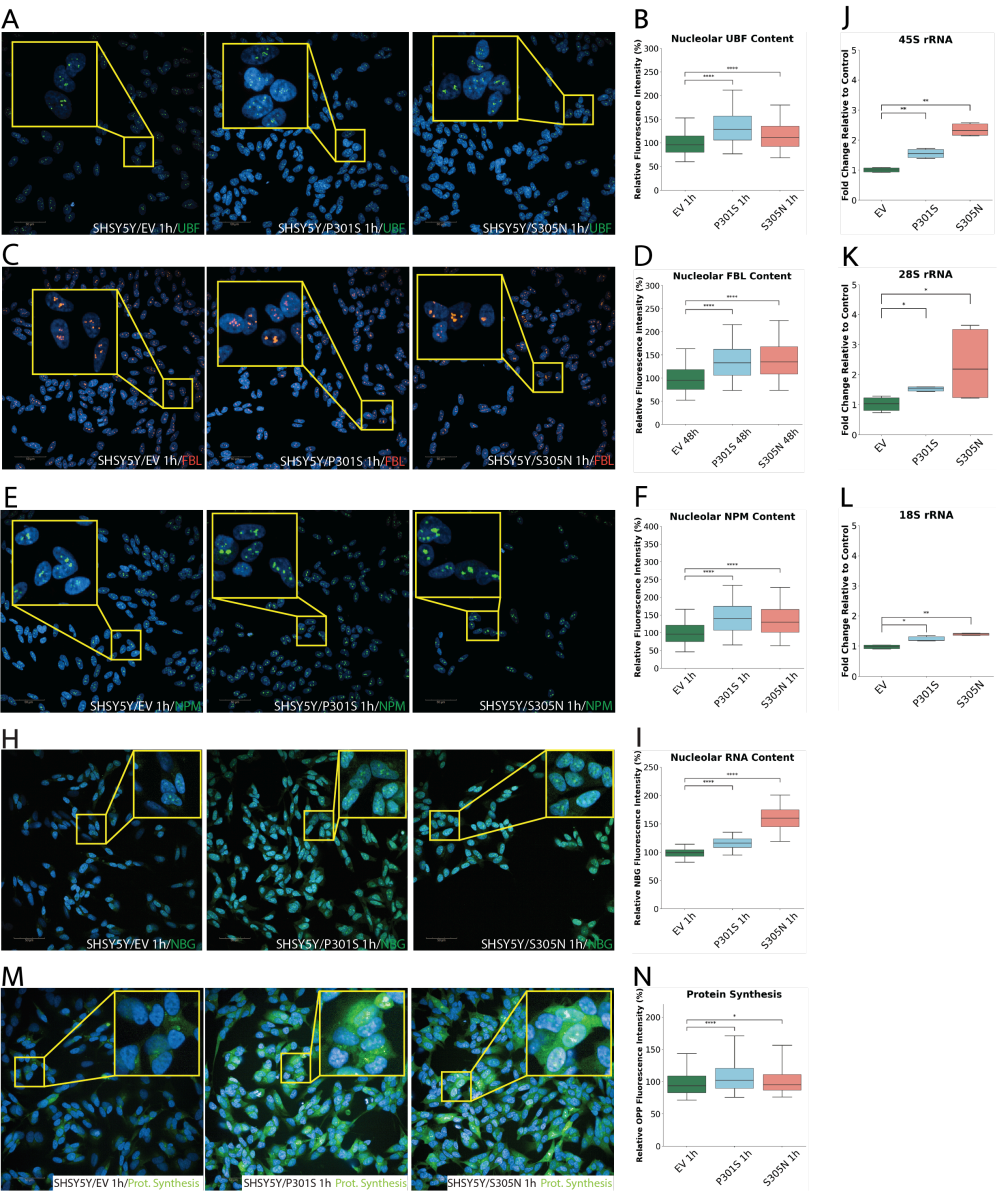
Mutant MAPT Expression Upregulates rDNA Transcription, Processing, and Nucleolar Activity in SH-SY5Y Cells. (A–F) Representative immunofluorescence images and corresponding quantifications of nucleolar protein content in tetracycline-induced SH-SY5Y cells expressing MAPT variants. (A–B) Upstream Binding Factor (UBF; green), a marker of the fibrillar center (FC) and key regulator of rDNA transcription, shows significantly increased nucleolar fluorescence intensity in both P301S and S305N cells relative to EV controls. (C–D) Fibrillarin (FBL; red), a marker of the dense fibrillar component (DFC) and essential for early rRNA processing, is markedly upregulated in mutant cells. (E–F) Nucleophosmin (NPM; green), a granular component (GC) protein involved in ribosome assembly, is also significantly elevated in both mutant lines. (H–I) Global nucleolar RNA content, assessed using Nucleolar Bright Green (NBG) dye, is significantly increased in mutant cells, indicating enhanced transcriptional activity. (J-L) qPCR analysis confirms upregulation of 45S pre-rRNA and its processed products (28S and 18S rRNA), indicating that MAPT mutations drive rDNA hyperactivation; transcript levels were normalized to β-actin and RSP27 and expressed as fold change relative to EV controls. (M–N) Nascent protein synthesis, measured by O-propargyl-puromycin (OPP) incorporation, shows a modest but significant increase in both mutant lines. Data represent mean ± SEM from ≥3 independent experiments, with ≥2,000 cells analyzed per condition and per experiment in high-content imaging experiments. Statistical comparisons were performed using two-sided Mann–Whitney U test with Bonferroni correction, following Shapiro–Wilk normality testing. *P < 0.05, **P < 0.01, ***P < 0.001, ****P < 0.0001. Scale bars: 50 μm.

The increased nucleolar accumulation of nP-Tau and enlargement of nucleoli observed in P301S and S305N cells prompted us to investigate whether these changes were due to enhanced nucleolar activity. The nucleolus is organized into three main sub-compartments: the fibrillar center (FC), where rDNA transcription takes place; the dense fibrillar component (DFC), where pre-rRNA processing occurs; and the granular component (GC), where pre-ribosomal subunit assembly is initiated ^51^. Each of these compartments is characterized by distinct protein markers commonly used in immunofluorescence studies: UBF, which regulates rDNA transcription and localizes to the FC; FBL, essential for early pre-rRNA processing and localized in the DFC; and NPM, involved in ribosomal maturation and localized to the GC. Using high-content immunofluorescence imaging of tetracycline-induced cells, we observed a consistent increase in the nucleolar signal intensity of all three markers in P301S and S305N lines. Specifically, UBF increased by ~ 35% in P301S and ~ 17% in S305N; FBL increased by ~ 61% in P301S and ~ 49% in S305N; and NPM increased by ~ 43% in P301S and ~ 36% in S305N, suggesting a global enhancement of nucleolar biosynthetic activity and structural remodelling (Fig. 2A–F). These increases persisted at 48 hours. Specifically, UBF rose to ~ 44% in P301S and ~ 46% in S305N; FBL remained elevated at ~ 37% and ~ 40% in P301S and S305N, respectively; and NPM continued to show elevated signal intensities with ~ 28% in P301S and ~ 35% in S305N compared to EV controls (Supp. Figure 1E-G). Together, these findings indicate that expression of *MAPT* mutants drives sustained and widespread upregulation of nucleolar biosynthetic machinery, affecting all three sub-compartments of the nucleolus.

To test the functional output of the nucleolus directly, we measured global nucleolar RNA levels using Nucleolar Bright Green (NBG) dye, which has high affinity for nucleolar RNAs (Fig. 2H). This revealed a significant increase in nucleolar RNA in the mutant lines, with mean intensities showing ~ 18% increase in P301S and ~ 60% increase in S305N relative to EV controls, indicating higher nucleolar RNA abundance (Fig. 2I). These elevations persisted and further increased at 48 hours, with P301S and S305N showing ~ 28% and ~ 75% increases, respectively, compared to EV controls (Supp. Figure 1H), indicating sustained nucleolar transcriptional output. Given that the nucleolus is a central hub for rRNA biogenesis, we next performed qPCR to assess transcriptional output by quantifying the expression of 45S pre-rRNA and its processed products, 28S and 18S rRNA. Consistent with the imaging results, we observed a robust upregulation of rRNA expression in both *MAPT* mutant lines (Fig. 2J–L). In P301S cells, there was approximately a 51% increase in 45S rRNA, a 43% increase in 28S rRNA, and a 37% increase in 18S rRNA compared to EV cells. In contrast, S305N cells demonstrated even more pronounced effects, with a 134% increase in 45S rRNA, a 145% increase in 28S rRNA, and a 47% increase in 18S rRNA relative to EV. These findings confirm that *MAPT* mutations lead to a hyperactivation of rDNA transcription and an increase in rRNA processing, showcasing enhanced nucleolar function as an early cellular phenotype.

Increased rRNA production supports ribosome biogenesis, which in turn drives protein synthesis. Given the increase in rRNA species observed, we wondered if this translated to increased protein synthesis. To do this, we measured nascently synthesis proteins in all cells following tetracycline induction using the Click-iT^™^ Plus OPP Alexa Fluor^™^ 488 Protein Synthesis Assay Kit (Fig. 2M). This method provides a fast, sensitive, and non-radioactive approach for detecting protein synthesis through the incorporation of O-propargyl-puromycin (OPP) into newly translated proteins. Our findings revealed a modest but significantly elevated level of newly synthesized proteins at 1 hour, with an approximate increase of ~ 8% in P301S and ~ 5% in S305N cells (Fig. 2N), suggesting a functional consequence of increased rRNA synthesis and processing. At 48 hours, this increase was further sustained in P301S (~ 11% vs. EV), while S305N showed only a marginal rise (~ 1.5%) that did not reach statistical significance (Supp. Figure 1I), indicating a possible divergence in translational output between the two mutants over time.

Together, these findings demonstrate that mutant *MAPT* enhances nucleolar rDNA transcription, rRNA processing, and protein synthesis in differentiated SH-SY5Y cells. While both P301S and S305N lines exhibit broadly similar trends, the magnitude and persistence of these effects vary, indicating a shared yet mutation-specific pattern of nucleolar upregulation.

### Mutant Tau Triggers p53 Signaling and Apoptosis

**Figure 3:**
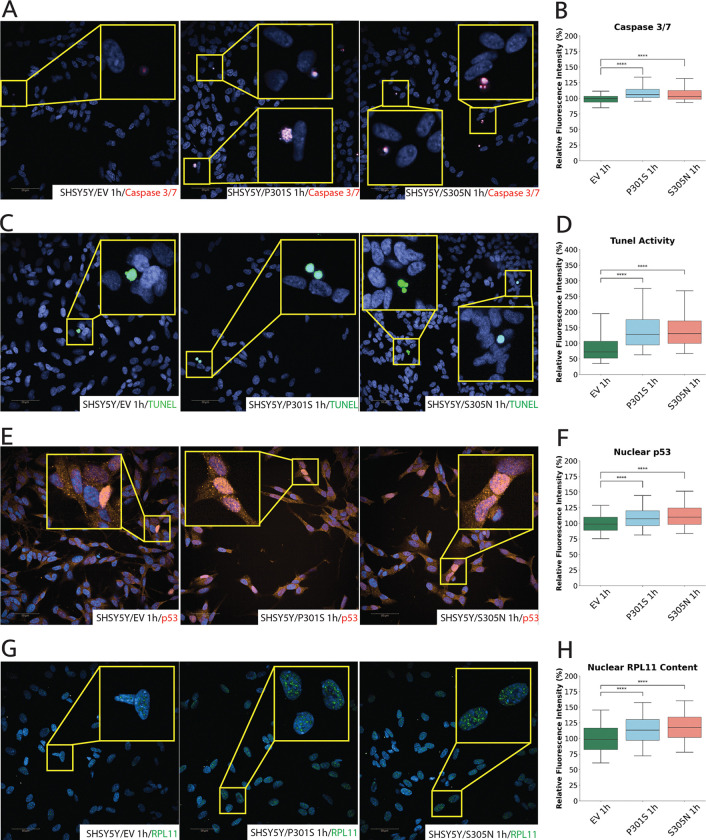
Mutant MAPT Expression Induces Nucleolar Stress and Apoptotic Signaling in SH-SY5Y Cells. (A–D) High-content immunofluorescence imaging and quantification of apoptotic markers in SH-SY5Y cells expressing MAPT mutants following tetracycline induction. (A–B) Caspase 3/7 activity (red), a key effector of apoptosis, is modestly but significantly elevated in both P301S and S305N cells compared to EV controls. (C–D) TUNEL assay (green), which detects DNA fragmentation, confirms increased apoptotic activity in mutant lines, consistent with early-stage cell death. (E–F) Nuclear p53 levels (red), a central stress response protein stabilized during nucleolar stress, are significantly upregulated in both mutant lines. (G–H) Immunolabeling of RPL11 (green), a ribosomal protein that participates in nucleolar stress, shows increased nuclear accumulation in P301S and S305N cells, supporting activation of the nucleolar stress response. Data represent mean ± SEM from ≥3 independent experiments, with ≥2,000 cells analyzed per condition and per experiment in most high-content imaging datasets. Statistical comparisons were performed using two-sided Mann–Whitney U test with Bonferroni correction, following Shapiro–Wilk normality testing. *P < 0.05, **P < 0.01, ***P < 0.001, ****P < 0.0001. Scale bars: 50 μm.

The nucleolus is a well-established sensor of cellular stress, and dysregulation of its function can lead to p53 stabilization and subsequent activation of apoptotic signalling cascades ^26^. Although the *MAPT* mutant cells exhibited elevated nucleolar activity, we consistently observed signs of cellular compromise during culture, not only in terms of altered morphology but also through a recurrent reduction in cell number across multiple high-content experiments, despite identical initial seeding densities. This prompted us to assess if this reduction in cell number is due to cell death. To achieve this, we used the CellEvent Caspase-3/7 Green ReadyProbes kit, a live-cell fluorogenic indicator that detects activated caspase-3/7 which is an early hallmark of apoptosis (Fig. 3A). This assay revealed a significant increase in caspase 3/7 activity, with an approximate ~ 9% increase in P301S and ~ 7% in S305N cells relative to EV (Fig. 3B). To independently validate apoptotic DNA damage, we employed the Click-iT Plus TUNEL Assay, which sensitively detects DNA fragmentation by incorporating a modified dUTP at the 3’-OH termini of fragmented DNA (Fig. 3C). In this assay, an alkyne-modified dUTP is incorporated during strand breaks, followed by a copper-catalyzed click reaction with Alexa Fluor 488-conjugated picolyl azide, enabling fluorescent detection. Using this method, we confirmed that mutant cells display elevated TUNEL positivity, with an approximate 54% increase in mean fluorescence intensity in both P301S and S305N cells relative to EV, indicating increased apoptotic DNA fragmentation (Fig. 3D). This apoptotic signal remained elevated at 48 hours in both lines indicating further cell death (Supp. Figure 1J).

Activation of p53 is one of the earliest outcomes of nucleolar stress and lies upstream of caspase activation in the apoptotic cascade. Moreover, p53 activation has been linked with tau pathology ^52,53^. To assess whether the increase in caspase activity and TUNEL positivity is due to p53 stabilisation, we used high-content imaging to quantify nuclear p53 levels in all tetracycline-treated SH-SY5Y cells (Fig. 3E). Immunolabeling revealed a significant increase in nuclear p53 fluorescence intensity in both P301S and S305N lines compared to EV controls, with an approximate ~ 10% increase in P301S and ~ 13% in S305N cells at 1 hour (Fig. 3F). These elevations persisted and further increased at 48 hours, with P301S and S305N showing ~ 13% and ~ 24% increases in nuclear p53 signal, respectively (Supp. Figure 1K), suggesting sustained and potentially amplifying p53 stabilization over time. While EV cells also exhibited low-level, background p53 signals, and in some cases elevated nuclear p53, this likely reflects sporadic basal stress responses within the population.

An increase in r-proteins, such as RPL11, is a defining feature of nucleolar stress. Under these conditions, RPL11 protein levels increases both at transcript and protein levels ^27^, translocating to the nucleoplasm, where it binds and inhibits MDM2, leading to the stabilization and nuclear accumulation of p53 ^54^. To determine whether the observed increase in p53 was linked to this mechanism, we immunostained for RPL11 in tetracycline-treated cells and quantified its nuclear levels (Fig. 3G). This revealed a significant increase in nuclear RPL11, with an approximate ~ 14% increase in P301S and ~ 18% in S305N mutant lines compared to EV (Fig. 3H), which remain sustained at 48h (Supp. Figure 1L), suggesting its upregulation as a consequence of nucleolar stress.

Together, these results demonstrate that expression of mutant MAPT variants initiates nucleolar stress, as evidenced by increased nucleoplasmic RPL11, which stabilizes p53 and activates the apoptotic cascade, linking tau-induced nucleolar dysfunction to early apoptotic signalling.

### Mutant Tau Induces Structural Expansion of Both Nuclear and Nucleolar Compartments in iPSC-Derived Neurons

**Figure 4:**
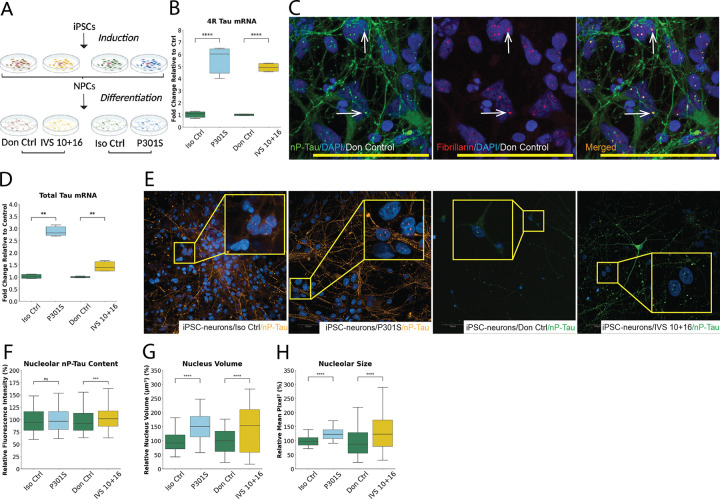
Mutant MAPT Expression Increases Nuclear and Nucleolar Volumes in iPSC-Derived Neurons (A) Schematic overview of the iPSC-to-neuron differentiation workflow highlighting four genotypes: isogenic control (Iso Ctrl), MAPT P301S mutant, donor control (Don Ctrl), and the intronic splicing variant (IVS 10+16). (B) Immunofluorescence images showing colocalization of non-phosphorylated Tau (nP-Tau, green) with the nucleolar marker fibrillarin (FBL; red) in DAPI-stained nuclei (blue) of iPSC-derived neurons from Don Ctrl. Arrows indicate nucleolar localisation of nP-Tau. qPCR analysis shows a significant increase in 4R tau (C) and total tau (D) transcript levels in both P301S and IVS 10+16 neurons relative to their respective controls, normalized to β-actin and RSP27 and shown as fold change. (E) Representative images display nuclear and nucleolar nP-Tau patterns across genotypes, with magnified insets. (F) Quantification of nucleolar nP-Tau intensity shows increases in both mutant lines. High-content morphometric analysis shows substantial increases in nuclear volume (G) and nucleolar size (H) in both P301S and IVS 10+16 neurons compared to their controls. Data represent mean ± SEM from ≥3 independent experiments with ≥1,000 cells per condition and per experiment. Statistical comparisons were performed using two-sided Mann–Whitney U test after Shapiro–Wilk normality testing. *P < 0.05, **P < 0.01, ***P < 0.001, ****P < 0.0001. Neurons from P301S and Iso Ctrl lines were analyzed at 75 days in culture, and neurons from Don Ctrl and IVS 10+16 lines at 50 days. Culture duration was calculated from the first day of NPC induction. Scale bars: 10 μm (B); 50 μm (E).

To evaluate the disease relevance of our findings in a more physiologically relevant context, we turned to induced pluripotent stem cells (iPSCs) to generate cortical-like neurons (Fig. 4A). We utilized iPSC lines representing four distinct genotypes. These included a line carrying the *MAPT* IVS 10 + 16 intronic mutation and donor-derived, non-isogenic control (Don Ctrl) ^48^, and a CRISPR-edited P301S *MAPT* mutant line along with its isogenic control (Iso Ctrl), both derived from a donor harbouring the *MAPT* P301L/WT background ^55^. These iPSCs were differentiated into neurons for either 50 days (IVS 10 + 16 and Don Ctrl) or 75 days (P301S and Iso Ctrl), measured from the onset of neural progenitor cell (NPC) induction. These time points were selected to capture the earliest detectable stage of mutant *MAPT* expression and assess its immediate impact on nucleolar structure and function. This early window of expression enables a direct comparison with the short-term, 1-hour induction paradigm used in the SH-SY5Y model. qPCR validation confirmed that both IVS 10 + 16 and P301S neurons expressed significantly elevated 4R tau transcript levels relative to their respective controls, with P301S neurons exhibiting more expression than the IVS 10 + 16 neurons, likely due to differences in their maturation (Fig. 4B). We first confirmed that nP-Tau is present in the nucleolus of these neurons, showing clear colocalization with the nucleolar marker FBL within the nuclei (Fig. 4C). This confirmed that nP-Tau exhibits nucleolar enrichment in human iPSC-derived neurons, consistent with our previous observations in SH-SY5Y cells and postmortem human brain tissue ^16^. Similarly, qPCR analysis for total tau revealed a similar increase in total tau expression in the mutant lines, similar to 4R tau (Fig. 4D).

High-content immunofluorescence analysis of differentiated neurons revealed distinct differences in nucleolar nP-Tau distribution across genotypes. While isogenic and donor controls showed baseline levels of nucleolar nP-Tau, both the P301S and IVS 10 + 16 neurons exhibited elevated signal intensities (Fig. 4E). Quantification revealed a modest (~ 1.5%) but statistically non-significant increase in nucleolar nP-Tau intensity in P301S neurons relative to isogenic controls (Fig. 4F), consistent with the nucleolar tau accumulation observed in SH-SY5Y cells (Fig. 1G). IVS 10 + 16 neurons showed a modest but statistically significant ~ 4% increase over donor controls. These findings indicate that even subtle early changes in *MAPT* expression can influence nucleolar tau accumulation, with some variation across mutations, as also observed in the SH-SY5Y model.

Given this increased nucleolar accumulation of nP-Tau, we next assessed nuclear and nucleolar morphometry to determine whether structural changes observed in SH-SY5Y cells were recapitulated in neurons. Nuclear volume analysis revealed a significant increase in both mutant lines relative to their controls, with P301S neurons exhibiting an approximate ~ 52% increase over isogenic controls, and IVS 10 + 16 neurons showing a ~ 45% increase compared to donor controls (Fig. 4G). Similarly, nucleolar size, measured via mean pixel area using FBL-based segmentation, was significantly increased in the mutant lines, with P301S neurons displaying a ~ 26% increase and IVS 10 + 16 neurons a ~ 35% increase relative to their respective controls (Fig. 4H). These findings parallel the increases in nuclear and nucleolar size observed in the SH-SY5Y model and further suggest that tau mutations drive consistent subnuclear structural remodelling across different neuronal systems.

Together, these results confirm that disease-associated *MAPT* mutations, both intronic (IVS 10 + 16) and coding (P301S), induce similar nuclear structural alterations in the iPSC-derived neurons as seen in the SH-SY5Y model.

### Mutant Tau Enhances Nucleolar rRNA Transcriptional Activity in iPSC-Derived Neurons

**Figure 5:**
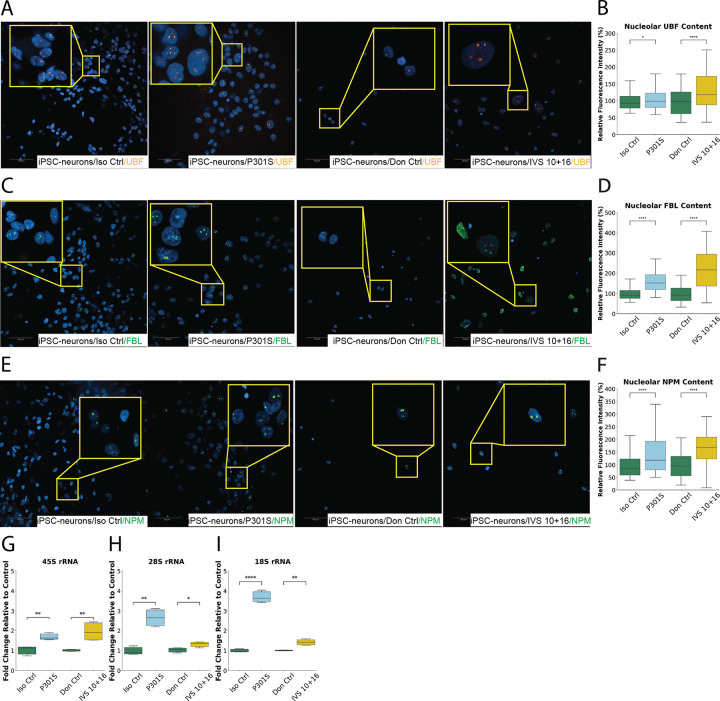
Mutant MAPT Expression Upregulates rDNA Transcription, Processing, and Nucleolar Activity in iPSC-Derived Neurons (A–F) Immunofluorescence images and quantitative analysis of nucleolar proteins in iPSC-derived neurons from four genotypes: isogenic control (Iso Ctrl), MAPT P301S mutant, donor control (Don Ctrl), and the MAPT IVS 10+16 intronic splicing variant. (A–B) Upstream Binding Factor (UBF; red), a marker of the fibrillar center (FC) involved in rDNA transcription, shows elevated nucleolar accumulation, particularly in IVS 10+16 neurons. (C–D) Fibrillarin (FBL; green), a dense fibrillar component (DFC) marker involved in rRNA processing, is significantly increased in both P301S and IVS 10+16 neurons; IVS 10+16 neurons also show diffuse nucleoplasmic FBL staining, a morphological hallmark of nucleolar stress. (E–F) Nucleophosmin (NPM; green), a granular component (GC) marker involved in ribosome assembly, displays significantly increased nucleolar localization in both mutant groups. qPCR analysis reveals significantly increased expression of 45S pre-rRNA (G), a marker of active rDNA transcription, and its processed products 28S (H) and 18S (I) rRNA in mutant lines. All expression levels were normalized to β-actin and RSP27 and presented as fold changes relative to respective control genotypes. Data represent mean ± SEM from ≥3 independent experiments, with ≥1,000 cells analyzed per genotype and per experiment. Statistical comparisons were performed using two-sided Mann–Whitney U test after Shapiro–Wilk normality testing. *P < 0.05, **P < 0.01, ***P < 0.001, ****P < 0.0001. Neurons from P301S and Iso Ctrl lines were analyzed at 75 days in culture, and neurons from Don Ctrl and IVS 10+16 lines at 50 days. Culture duration is calculated from the first day of NPC induction. Scale bars: 50 μm.

To assess whether the transcriptional changes observed in the differentiated SH-SY5Y cells also occur in a more disease-relevant system, we examined the iPSC-derived neurons expressing *MAPT* mutations. We focused on evaluating nucleolar structural and functional markers representing the FC (UBF), DFC (FBL) and GC (NPM). High-content immunofluorescence imaging revealed increased nucleolar signals of all three markers in both P301S and IVS 10 + 16 neurons compared to their respective controls (Fig. 5A–F). Specifically, quantitative analysis showed that UBF increased modestly by ~ 3% in P301S neurons and by ~ 33% in IVS 10 + 16 neurons (Fig. 5B). FBL showed a more robust increase of ~ 61% in P301S and an even greater ~ 124% in IVS 10 + 16 neurons (Fig. 5D). Similarly, NPM levels rose by ~ 48% in P301S and ~ 67% in IVS 10 + 16 neurons (Fig. 5F). These findings indicate a marked enhancement of nucleolar biosynthetic activity, particularly in the steps of rRNA generation and processing and ribosomal maturation.

To directly assess functional output, we next performed qPCR to measure the expression of 45S pre-rRNA and its processed products 28S and 18S rRNAs. The analysis confirmed significant upregulation of all three rRNA species in the P301S and IVS 10 + 16 neurons relative to their respective controls (Fig. 5G–I), suggesting enhanced rDNA transcription and rRNA processing. Specifically, P301S neurons exhibited a ~ 60% increase in 45S, ~ 141% increase in 28S, and ~ 283% increase in 18S rRNA compared to their isogenic controls. Similarly, IVS 10 + 16 iPSC-derived neurons showed ~ 103% higher 45S, ~ 30% higher 28S, and ~ 47% higher 18S levels compared to donor-matched controls. These transcriptional elevations strongly support a mutation-driven enhancement of nucleolar activity.

Together, these findings demonstrate that *MAPT* mutations stimulate rDNA transcription and downstream rRNA processing in iPSC-derived neurons, mirroring the effects observed in SH-SY5Y cells. This pattern supports a model in which mutant tau drives nucleolar hyperactivation, not only at the transcriptional level but also through amplification of post-transcriptional biosynthetic machinery, across both transformed and patient-derived neuronal systems.

### Mutant Tau Triggers Nucleolar Stress and Apoptotic Signaling in iPSC-Derived Neurons

**Figure 6:**
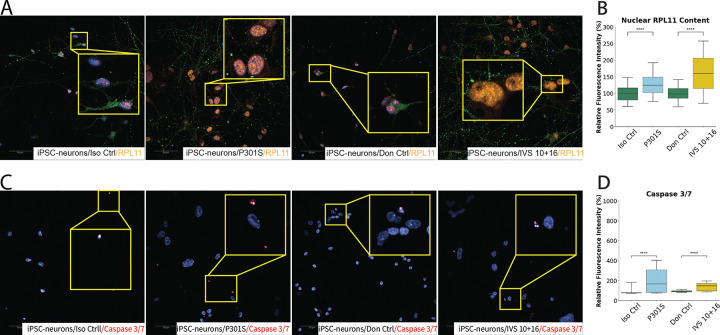
Mutant MAPT Expression Induces Nucleolar Stress and Apoptotic Signalling in iPSC-Derived Neurons (A–B) Immunofluorescence imaging and quantification of nucleolar stress and apoptosis markers in iPSC-derived neurons from isogenic control (Iso Ctrl), MAPT P301S mutant, donor control (Don Ctrl), and MAPT IVS 10+16 splicing variant lines. (A–B) RPL11 (orange), a ribosomal protein that accumulates in the nucleoplasm under nucleolar stress, displays markedly increased nucleoplasmic localization in neurons from P301S and IVS 10+16 lines. Cell morphologies were visualized using nP-Tau (green). (C–D) Caspase 3/7 activation (red, within DAPI stained nuclei), a marker of apoptosis, is significantly elevated in P301S and IVS 10+16 neurons compared to controls. Data represent mean ± SEM from ≥3 independent experiments, with a minimum of 1,000 cells analyzed per genotype, per experiment. *P < 0.05, **P < 0.01, ***P < 0.001, ****P < 0.0001. Neurons from P301S and Iso Ctrl lines were analyzed at 75 days in culture, and neurons from Don Ctrl and IVS 10+16 lines at 50 days. Culture duration is calculated from the first day of NPC induction to the day of analysis. Scale bars: 50 μm.

To evaluate whether nucleolar stress underlies the cellular compromise observed in iPSC-derived neurons expressing mutant tau, we first examined the distribution of RPL11 which is involved in p53 stabilization during nucleolar stress. High-content immunofluorescence imaging revealed increased nuclear RPL11 levels in both P301S and IVS 10 + 16 neurons compared to their respective controls (Fig. 6A). Quantitative analysis showed a ~ 28% increase in nuclear RPL11 fluorescence in P301S neurons and a more robust ~ 62% increase in IVS 10 + 16 neurons relative to controls (Fig. 6B). These increases suggest a redistribution of RPL11 into the nucleoplasm, a hallmark of nucleolar stress. Consistent with this, we also observed occasional diffuse nucleoplasmic FBL staining in IVS 10 + 16 neurons (Fig. 5C) – a well-recognized morphological signature of nucleolar stress, further supporting activation of this stress pathway in the mutant lines.

Similar to our observation in SH-SY5Y cells showing that mutant tau induced apoptotic signalling, we next assessed caspase 3/7 activity in iPSC-derived neurons. High-content imaging revealed a significant increase in caspase 3/7 signal in both mutant lines (Fig. 6C). Specifically, P301S neurons showed an approximate ~ 105% increase compared to their isogenic controls, while IVS 10 + 16 neurons exhibited a ~ 54% increase relative to donor-matched controls (Fig. 6D). These findings reinforce the notion that MAPT mutations promote apoptosis, as a downstream consequence of persistent nucleolar stress.

Together, these findings demonstrate that mutant tau initiates a nucleolar stress response in human iPSC-derived neurons similar to the SH-SY5Y cells, culminating in caspase-mediated apoptosis.

## Discussion

Since its discovery ^1^, tau has evolved from being seen primarily as a microtubule-associated protein to a multi-functional protein with a critical nuclear function ^39,49^. Our findings add to this evolving understanding by revealing that disease-associated *MAPT* mutations trigger a cascade of nucleolar changes in human neuronal models, marked by increased nucleolar size and activity driven by rDNA hyperactivity, resulting in nucleolar stress, and subsequent apoptotic signalling. These effects are conserved across both inducible SH-SY5Y cells and iPSC-derived neurons, suggesting that nucleolar dysfunction may represent a shared, early pathogenic mechanism in tauopathies caused by *MAPT* mutations.

### Tau and the Nucleolus: Revisiting a Neglected Interaction

Historically, immunolocalization studies from the late 1990s by Lester (Skip) Binder’s group reported nuclear tau in neurons, specifically showing that non-phosphorylated tau (nP-Tau), recognized by the Tau-1 antibody which detects tau dephosphorylated at serine residues 195, 198, 199, and 202, localizes to the fibrillar component of the nucleolus ^19–22^. Indeed, the nucleolar pool of tau appears to be majorly non-phosphorylated, hinting at compartment-specific post-translational modifications that could modulate its function. Given tau’s DNA-binding properties ^13^, its localization in the nucleolus suggests potential roles in rDNA transcriptional regulation, chromatin remodeling, or safeguarding ribosomal gene integrity ^39^. In support of this, more recently, we revealed tau’s localisation to the nucleolus in various cell types and the human brain, revealing that nP-Tau associated with the nucleolar remodelling complex helping to regulate rDNA transcription ^16^, implying an intentional and regulated nuclear function. Moreover, tau has been found to influence chromatin dynamics ^10–12,56^ and DNA integrity ^13,14^. The current work builds on this by showing that nP-Tau not only localizes to the nucleolus in iPSC-derived neurons but also accumulates there upon the expression of mutant tau in different *MAPT* mutants similar to the accumulation of UBF, FBL and NPM and associated with an increase in nucleolar size and activity. This further confirms our initial observations ^16,40^ in suggesting that tau may act as a regulator of nucleolar structure and function similar to canonical nucleolar proteins. These insights challenge the dogma that tau’s primary function is cytoskeletal and highlight the importance of tau–nuclear interactions for deep understanding of the mechanism of neurodegeneration in tauopathies.

### Nucleolar Hypertrophy in Neurodegenerative Diseases: Adaptive or Pathological?

The nucleolus, classically viewed as the cellular factory for ribosome production is now recognised as a dynamic sensor and regulator of cellular stress, growth, and survival ^51^. In the context of neurodegeneration, alterations in nucleolar size, morphology, and function have increasingly been recognized as either early disease markers or downstream responses. Here, we find that the *MAPT* mutants in both SH-SY5Y cells and iPSC-derived neurons were associated with pronounced nucleolar hypertrophy, evident by increased nucleolar volume and upregulation of nucleolar proteins (UBF, FBL, NPM1), alongside augmented rDNA transcription and processing to 28S, and 18S rRNA species. This aligns with the hypothesis that tau mutations aberrantly induce nucleolar activity and alter nuclear architecture in ways that can initially be compensatory but ultimately become maladaptive. Evidence for nucleolar hypertrophy as an early marker in neurodegenerative diseases has been observed by several studies. For example, many studies from the Troncoso lab have described significant nucleolar hypertrophy in several brain regions in asymptomatic but not AD brain samples ^57–59^. More recently, work from the Galas group similarly reported nucleolar hypertrophy in early-stage tau pathology in the Tau22 mice model, which was accompanied with nucleolar shrinkage in later stage of the tau pathology ^60^. Similarly, Mizielinska et al. (2017) demonstrated a bidirectional pattern of nucleolar remodelling in C9orf72-associated FTLD, where nucleolar volume was significantly enlarged in neurons harboring poly(GR) inclusions or RNA foci, but reduced overall across the patient cortex, suggesting that nucleolar hypertrophy may occur transiently in response to toxic aggregates before declining in late-stage pathology ^34^. Taken together, these studies argue that nucleolar hypertrophy may represent a core early molecular hallmark across multiple neurodegenerative diseases. While initially aimed at boosting ribosome biogenesis and sustaining translation under stress, prolonged nucleolar enlargement may lead to deleterious outcomes.

Indeed, perturbations in nucleolar function can trigger a conserved response termed “nucleolar stress,” leading to p53 stabilization and, if unresolved, apoptosis ^26,61^. In both SH-SY5Y cells and iPSC-derived neurons, we noticed several signs of nucleolar stress. Notably, FBL, often redistributed in response to such stress ^26^, showed diffuse nucleoplasmic staining in IVS 10 + 16 neurons, a morphological hallmark of nucleolar destabilization. We have previously shown that this redistribution is associated with tau dysfunction and chromatin alterations ^16,40^, further implicating mutant tau in disrupting nucleolar integrity. Another key indicator of nucleolar stress is RPL11, a ribosomal protein that translocates from the nucleolus to the nucleoplasm upon stress and binds MDM2, the negative regulator of p53, thereby stabilizing p53 and triggering downstream cell death pathways ^26,62^. Our data show increased RPL11 accumulation in all the *MAPT* mutant lines in both SH-SY5Y cells and iPSC-derived neurons, alongside a marked increase in nuclear p53 signal. This supports the canonical RPL11-MDM2-p53 axis as a mechanistic link between nucleolar remodeling and apoptosis in our model.

Pertinent to this, recent evidence using both in vitro starvation and in vivo TBI models demonstrated that nuclear accumulation of phosphorylated tau results in significant increase in p53 stabilization, cleaved caspase-3 levels and TUNEL positive neurons, suggesting that nuclear tau mislocalization alone can prime neurons for apoptotic cascades ^53^. The authors revealed that this was associated with fibrillarin dispersion and alteration of nucleolar morphology – classic hallmarks of nucleolar stress. Similarly, in this study, we find that the increase in p53 was accompanied by elevated caspase 3/7 activation, confirming that the nucleolar stress is not merely an adaptive response but proceeds toward apoptosis. In sum, our findings indicate that *MAPT* mutations disrupt nucleolar homeostasis by triggering rDNA transcriptional hyperactivation and structural remodelling, which initiate a cascade of nucleolar stress responses involving RPL11 and p53, ultimately culminating in caspase-mediated apoptosis. This mechanistic chain provides a compelling framework that connects tau mutations to nucleolar dysfunction and neuronal loss.

Altogether, our findings propose a model where tau-induced rDNA hyperactivation and rRNA processing, leads to nucleolar stress, culminating in apoptosis. Further work is required to confirm if this mechanism is universal across other *MAPT* mutations and tauopathies and to dissect whether the effects of tau on the nucleolus are direct or secondary to broader transcriptomic dysregulation. Nonetheless, our results position the nucleolus as a previously underappreciated site of tau toxicity and a potentially valuable therapeutic target.

## Supplementary Material

Supplementary Files

This is a list of supplementary files associated with this preprint. Click to download.

• SuppFigure1MAPTMuhammadetalMAPTNucleolarDysfunctionv1.png

• TauPaperTable1.docx

• TauPaperTable2.docx
